# Magnetic surface molecularly imprinted poly(3-aminophenylboronic acid) for selective capture and determination of diethylstilbestrol

**DOI:** 10.1039/c8ra01250d

**Published:** 2018-04-09

**Authors:** Wen-Rui Zhao, Tian-Fang Kang, Li-Ping Lu, Shui-Yuan Cheng

**Affiliations:** Key Laboratory of Beijing on Regional Air Pollution Control, College of Environmental and Energy Engineering, Beijing University of Technology Beijing 100124 P. R. China kangtf@bjut.edu.cn kangtf@sina.cn +86 10 67391983 +86 10 67391659

## Abstract

Diethylstilbestrol (DES) is considered a representative example of an exogenous endocrine disrupting compound (EDC). It can retard development in infants, lead to serious metabolic regulation disorders, and even result in distortion and cancer in the reproductive system. Therefore, achieving rapid and accurate analysis of trace amounts of DES in complex environments is of great importance to human health and for environmental protection. Novel magnetic molecularly imprinted polymers (MIPs) with excellent molecular recognition ability and super water-compatibility were developed for the selective capture of DES in water samples. Fe_3_O_4_@SiO_2_ magnetic nanoparticles (NPs) were synthesized and used as support cores. Molecularly imprinted poly(3-aminophenylboronic acid) (poly(APBA)), synthesized on magnetic cores based on a surface-imprinting strategy, can preferentially bind DES molecules in water samples. The magnetic core–shell MIPs (denoted as Fe_3_O_4_@SiO_2_@APBA/MIPs) exhibited high binding capacity and favorable recognition specificity for DES in water. The adsorption kinetics and experimental isotherm data of DES on magnetic MIPs can be well described by the pseudo-second-order kinetic model and the Langmuir isotherm, respectively. The imprinted nanoparticles were subjected to magnetic solid-phase extraction (MSPE) of DES from water samples. The DES content in the samples was determined by high-performance liquid chromatography (HPLC). The peak area increased linearly with increasing DES concentration over the range 0.08–150 μg L^−1^, with a detection limit of 0.03 μg L^−1^. The recoveries for spiked lake water samples were in the range 97.1–103.2%, with relative standard deviation (RSD) of 2.8–4.3% (*n* = 6).

## Abstract 2

Imprinted poly(APBA) nanoshell on Fe_3_O_4_@SiO_2_ surface was first synthesized and used for MSPE of diethylstilbestrol followed by HPLC determination.

## Graphical abstract

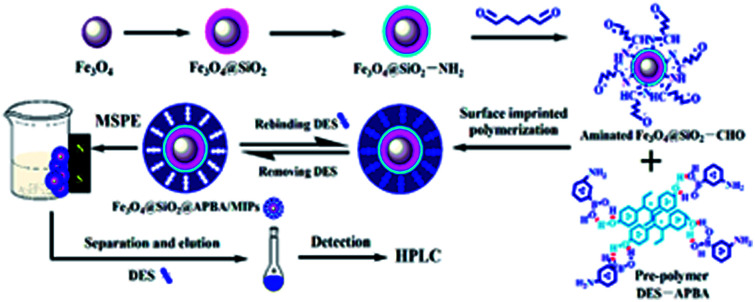

## 1. Introduction

Diethylstilbestrol (DES) is considered a representative example of an exogenous endocrine disrupting compound (EDC), which directly interferes with the endocrine function by simulating or antagonizing the normal endogenous hormones.^1^ DES can be bio-accumulated in the food chain and remain in organisms for a long time, having a serious impact on the organism even at very low concentrations. Once the human body is exposed to DES, the secretion and transport of natural hormones will be destroyed. DES can retard development in infants, lead to serious metabolic regulation disorders, and even result in distortion and cancer in the reproductive system.^1,2^ In recent years, there has been a dramatic increase in the use of hormonal cosmetics and drugs, while DES has been misused in order to promote the growth of animals. Through use of liquid chromatography coupled with mass spectrometry (LC-MS), researchers have found DES residues in foods (*e.g.* eggs, meat, and milk), as well as in the soil and water environment.^3^ Currently, the use of DES is banned or restricted in China, the United States, and in many countries of Europe. The development of new technologies for the detection DES has also been a matter of international concern. Therefore, achieving rapid and accurate analysis of trace amounts of DES in complex environments is of great importance to human health and for environmental protection.

Numerous methods have been used for the detection of DES in water, including LC-MS,^3^ gas chromatography coupled with mass spectrometry (GC-MS),^4^ high-performance liquid chromatography (HPLC) equipped with diode-array detector (HPLC-DAD),^5,6^ immunoassay,^7^ and capillary electrochromatography.^8^ Due to the generally low concentration of DES in real environmental samples, high-performance detection using these traditional methods usually requires an efficient sample preparation step for rapid pre-concentration, such as solid-phase extraction, liquid-phase extraction, and liquid-phase microextraction. The main challenges associated with these techniques for DES determination are poor selectivity and low recovery.^5,6^ However, by using magnetic molecularly imprinted polymer (MMIP) particles as the solid-phase extraction agent, DES can not only be selectively extracted from water samples, but also separated quickly under an external magnetic field. Therefore, the pretreatment process can be performed quickly and easily.

In general, MIPs are synthesized using templates, with a suitable monomer and cross-linking agent, with an initiator to initiate the polymerization. The template molecules are then removed to create recognition cavities with many functional recognition sites. These cavities can match the size, shape, and spatial structure of the template molecule. Thus, MIPs with a specific ability for molecular recognition can selectively rebind the target. MIPs have been widely used in sensors,^9^ and for catalysis,^10^ separation, and purification.^11^ Recent studies have focused on the preparation of MIPs for the enriching and detection of DES, bisphenol A (BPA), or other estrogens^6,12,13^ as well as an evaluation of MIPs toward DES in the organic phase due to their excellent adsorption properties.^1,6,13–17^ In particular, the application of MIPs in the construction of sensors with a high affinity and selectivity for the target is highly promising. Recently, a series of novel electrochemical sensors combined MIPs with various new nano-materials, and excellent performances have been reported.^18–22^ The application of MIPs for the detection of trace amounts of DES in water is particularly promising.

It is difficult to synthesize MIPs directly in the aqueous phase because the formation of hydrogen bonds between the template and the functional monomer can be easily interfered with by water molecules.^23^ Furthermore, template molecules of estrogens have poor solubility in the aqueous phase. In order to improve the water-compatibility of MIPs, Wu *et al.* grafted hydrophilic 2-hydroxyethyl methacrylate brushes onto the surface of the MIPs.^24^ Other typical methods include the use of hydrophilic functional monomers, such as α-methacrylic acid,^1,17^ 2-acrylamido-2-methylpropanesulfonic acid,^25,26^ 4-vinylpyridine,^25^ acryloyl-β-cyclodextrin,^27,28^ and 3-aminophenylboronic acid,^29^ in the synthesis of the MIPs. These methods are simple and can improve the surface hydrophilicity of the MIPs. As a water-soluble functional monomer, aminophenylboronic acid (APBA) can be used to prepare MIPs of DES due to the presence of multiple functional groups including amino, hydroxyl, and phenyl groups, and can be polymerized both in aqueous and organic phase solution. Thin film polymers of APBA (poly(APBA)) have been used as the coating substrate on solid supports such as polystyrene nanoparticles (NPs), microspheres, and the gold surface of quartz crystal microbalance electrodes.^29–31^

MMIPs^15,17^ can be prepared by synthesizing MIPs on the surface of Fe_3_O_4_ magnetic nanoparticles. Therefore, MMIPs can not only specially capture target molecules, but can also be rapidly magnetically separated from the solution. There are many methods for the synthesis of MMIPs, such as emulsion polymerization, the sol–gel method, and suspension polymerization, for example. In general, the magnetic properties of MIP microspheres obtained by traditional emulsion polymerization are usually weak because of the nucleation of micelles leading to low encapsulation efficiency.^32^ The imprinted film on particles synthesized by the sol–gel method, the residue of the hydrophobic portion of the silane coupling agent, is hard to avoid resulting in adhesion between the particles.^26,33^ However, suspension polymerization with water as the continuous phase can be used to prepare imprinted nanoparticles with a small particle size, strong magnetic properties, and good dispersion in the aqueous phase.^12^

**Fig. 1:**
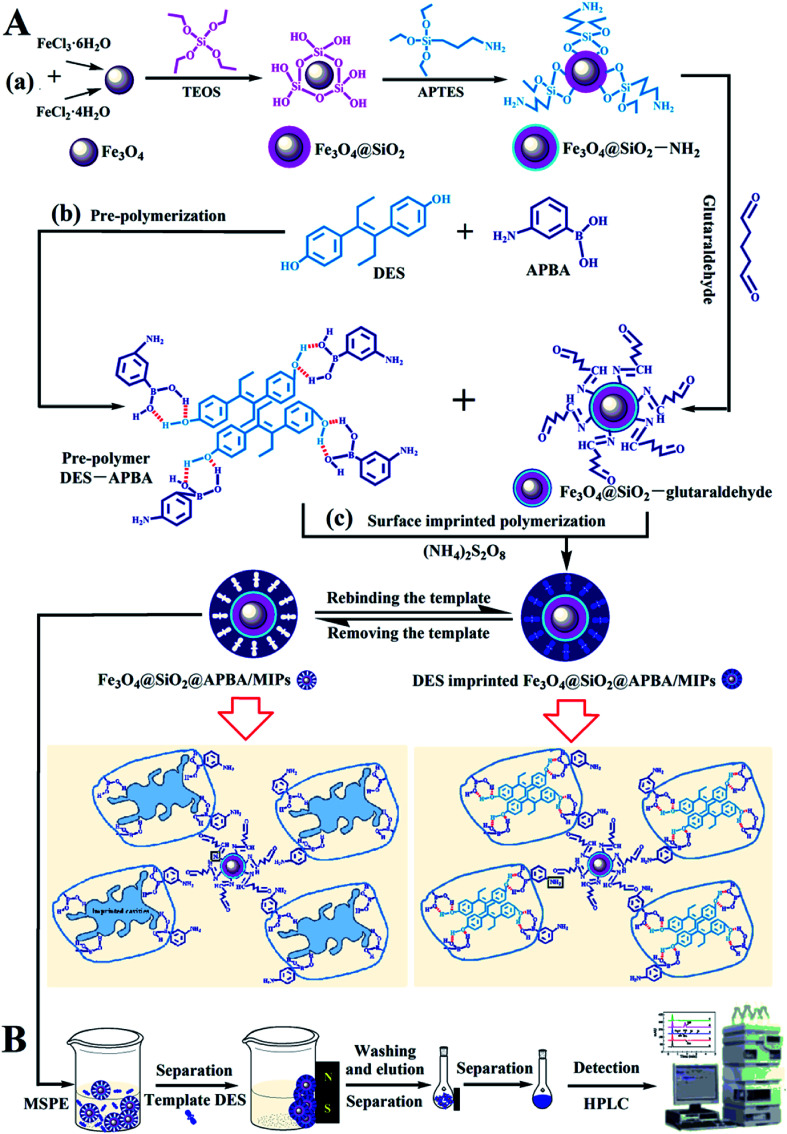
Schematic procedure for (A) the preparation of DES-imprinted MIPs and (B) application to MIPs-MSPE-HPLC method for detection of DES.

In this study, we developed super water-soluble DES-imprinted MMIPs (Fe_3_O_4_@SiO_2_@APBA/MIPs) with a multilayer core–shell structure for the selective recognition and extraction of DES from the aqueous phase. APBA was used as the hydrophilic monomer and cross-linking reagent. The binding properties, including molecular binding capacity and specific recognition ability, were investigated in detail. The MIP NPs exhibited much higher binding capability for DES in water than previously reported.^5,27^ The MIPs as adsorbents were used in enriching trace DES from lake water samples by magnetic solid-phase extraction (MSPE). The preparation procedure and working principle of Fe_3_O_4_@SiO_2_@APBA/MIPs (Fig. 1A) and their applications to MSPE-HPLC (Fig. 1B) are schematically illustrated in Fig. 1.

## 2. Experimental

### 2.1 Reagents

DES, APBA, 3-aminopropyl triethoxy silane (APTES), and tetraethyl orthosilicate (TEOS) were purchased from J&K Scientific Ltd. Glutaraldehyde (GA), FeCl_3_·6H_2_O, FeCl_2_·4H_2_O, potassium peroxydisulfate, and other reagents were obtained from Beijing Chemical Reagent Company (Beijing, China). Bisphenol A was from Chengdu Xiya Chemical Co., Ltd. (Chengdu, China). Phenol, bisphenol F, estrone, and estradiol were provided by Tianjin Chemical Reagent Co. Ltd (Tianjing, China). All reagents were of analytical grade. Acetonitrile (ACN) for HPLC was of HPLC-reagent grade and was supplied by J&K Scientific Ltd. (Beijing, China). All solutions were prepared with ultrapure water (Milli-Q Advantage A10 Water Purification System, Millipore Corporation, France). DES (100 mg) was dissolved in 100 mL of ethanol for the preparation of 1000 mg L^−1^ of DES stock solution, and stored at 4 °C until use. DES solutions with required concentration could be diluted with ultrapure water for further use. The elution solution was a mixture of methanol-0.1 M acetic acid (5.0 mL, v/v, 9/1).

### 2.2 Instruments

Scanning electron microscopy (SEM) and transmission electron microscopy (TEM) images of functionalized Fe_3_O_4_ NPs were obtained by SU-8010 (Hitachi) and HT7700 (Hitachi), respectively. X-ray energy dispersive spectroscopy (EDS) was used to obtain the chemical composition of the samples. Fourier transform infrared (FTIR) spectra were recorded on an IR Prestige-21 FTIR spectrometer (Shimadzu). The thermal stability of the imprinted NPs was analyzed using a TG 209 F3 thermogravimetric analyzer (TGA; Netzsch, Germany) at a heating rate of 10 °C min^−1^ under an air atmosphere. Magnetization measurements of magnetic nanoparticles were performed using a vibration sample magnetometer (VSM; Lake Shore 7410). The static water contact angles (CA) of functionalized Fe_3_O_4_ NPs were measured using an OCA 15 Pro video optical measurement instrument of CA (Data Physic, Germany) with 2.5 μL of deionized water droplets. Spectrophotometric experiments were carried out using a UV-2450 spectrophotometer (Shimadzu). Chromatographic analyses were performed using a Model 1260 HPLC instrument (Agilent Technologies Co., Ltd., USA), mainly equipped with a diode-array detector and a chromatographic column (150 mm × 4.6 mm C_18_). Optimized HPLC conditions were injection volume, 20 μL; mobile phase, acetonitrile/ultrapure water (6 : 4, v/v); flow rate, 0.8 mL min^−1^; temperature of the column, 25 °C; DAD detection wavelength, 240 nm.

### 2.3 Preparation of functionalized Fe_3_O_4_@SiO_2_ NPs

Magnetic Fe_3_O_4_ NPs were synthesized according to the coprecipitation method that we reported previously.^34^ In brief, a mixture of FeCl_2_·4H_2_O (2.0 g), FeCl_3_·6H_2_O (5.2 g), 12 M HCl (0.85 mL), and 25 mL of water was degassed with high-pure nitrogen with stirring before use. Then, the mixed solution was added dropwise into 250 mL of 1.5 M NaOH solution in a water bath at 80 °C, and was stirred for 1 h under N_2_ protection. After cooling down, the obtained Fe_3_O_4_ NPs were washed repeatedly five times with water and ethanol, and then collected magnetically, before being dried under an N_2_ atmosphere.

Fe_3_O_4_@SiO_2_ NPs functionalized with amino-groups were prepared based the Stöber process,^24,35,36^ with minor modifications. As-prepared Fe_3_O_4_ NPs (100 mg) were dispersed in a mixture of ethanol and ultrapure water (180 mL, 8 : 1, v/v) and ultrasonicated for 15 min. Then, 1.0 mL of ammonia aqueous solution (28%, w/w) was added into the suspension under vigorous stirring for 30 min in a water bath at 30 °C. After adding 1 mL of TEOS dropwise, the reaction proceeded continuously for 45 min; then, 0.5 mL aminopropyltriethoxysilane (APTES) was added dropwise into the suspension. The reaction between Fe_3_O_4_@SiO_2_ NPs and APTES lasted for 4 h at 30 °C. The obtained Fe_3_O_4_@SiO_2_–NH_2_ NPs were collected using a magnet and washed with ethanol and ultrapure water three times, followed by drying under nitrogen gas protection overnight.

Fe_3_O_4_@SiO_2_–NH_2_ NPs were modified with glutaraldehyde^12^ as the bridging agent to introduce free aldehyde groups for further covalent anchoring of MIPs grafted tightly on the surface of the support substrates. Briefly, 50 mg of Fe_3_O_4_@SiO_2_–NH_2_ NPs was dispersed in 50 mL of excess glutaraldehyde aqueous solution (5%, v/v) with slow stirring to form a homogeneous suspension and allowed to react for 12 h at room temperature under continuous stirring. It is necessary here to ensure that the concentration and volume of the glutaraldehyde solution are sufficiently in excess, and the magnetic particles are added into the glutaraldehyde solution. The order cannot be reversed to avoid aminated nanoparticles from being cross-linked by an insufficient amount of glutaraldehyde. The obtained Fe_3_O_4_@SiO_2_–glutaraldehyde NPs were magnetically separated and then rinsed with equal volumes of ultrapure water three times, and finally collected magnetically.

### 2.4 Preparation of water-compatible MMIPs

The DES imprinted water-compatible magnetic MIPs (denoted as Fe_3_O_4_@SiO_2_@APBA/MIPs) were prepared *via* a surface-imprinting polymerization process.^30,37^ The Fe_3_O_4_@SiO_2_–glutaraldehyde NPs were redispersed in 50 mL of 20 mM APBA aqueous solution, stirred for 30 minutes, and statically aged for 12 h to allow self-assembly on the Fe_3_O_4_@SiO_2_ NPs surface. For prepolymerization, 50 mL of the template-monomer solution containing 20 mM APBA and 5 mM DES was shaken for 30 min at room temperature and set aside for 12 h. Then, the self-assembly suspension and 20 mg of K_2_S_2_O_8_ were added. The mixture was stirred at reflux at 60 °C for 24 h under an N_2_ atmosphere for polymerization of poly(APBA). After magnetic separation, the obtained DES-loaded MIPs (denoted as Fe_3_O_4_@SiO_2_@APBA/MIPs-DES) were rinsed with ethanol and ultrapure water and then eluted with the mixture of methanol-0.1 M acetic acid (5 mL, 9 : 1, v/v) repeatedly with shaking to remove DES, until the eluent was free from DES as detected by UV-vis spectrometry at 240 nm.^12,24^ Finally, the resulting Fe_3_O_4_@SiO_2_@APBA/MIPs were washed thoroughly with ethanol and ultrapure water and dried at 40 °C under nitrogen gas protection overnight. Thus, recognition cavities complementary to DES in shape, size, and chemical functionality were formed in imprinted layers, which could selectively rebind DES. For comparison, non-imprinted polymers (Fe_3_O_4_@SiO_2_@APBA/NIPs) were prepared using the same procedures in the absence of DES.

### 2.5 Adsorption experiments

Static adsorption experiments were performed at 288, 293, 298, 308, and 318 K to investigate the effect of temperature on the adsorption capacities of Fe_3_O_4_@SiO_2_@APBA/MIPs toward DES. MIPs or NIPs (20.0 mg) were suspended in a series of 50 mL DES aqueous solutions with various initial concentrations (*C*_0_, mg L^−1^) ranging from 0.0500 to 100 mg L^−1^. After a series of adsorbent–adsorssbate mixtures were mechanically shaken for 3 h at different temperatures, the MIP or NIP NPs were separated magnetically, and then the equilibrium adsorption concentration of DES (*C*_e_, mg L^−1^) in the collected supernatant was measured by UV-vis spectrophotometer operating at 240 nm. The binding amounts of DES on MIPs or NIPs at equilibrium, defined as the equilibrium adsorption capacity (*Q*_e_, mg g^−1^), could be calculated using eqn (1):^25^1
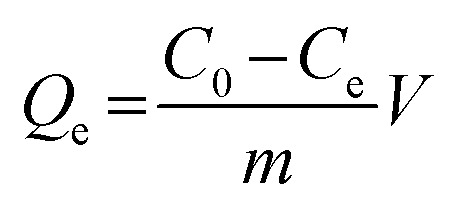
where *V* (L) represents the volume of DES solution and *m* (g) denotes the mass of Fe_3_O_4_@SiO_2_@APBA/MIPs (or NIPs) used.

The binding kinetics experiment procedure was similar to the static adsorption study for the monitoring of the minimum adsorption equilibrium time. Fe_3_O_4_@SiO_2_@APBA/MIPs or NIPs (20.0 mg) were added to 50 mL of DES solution with an initial concentration (*C*_0_) of 60 mg L^−1^. The suspension was shaken continuously for a series of time intervals (*t*) from 5 to 200 min at 298 K. The temporal concentration of DES (*C*_t_, mg L^−1^) in the supernatants was analyzed by UV. The binding amounts for DES with different contact time *t*, defined as the temporal adsorption capacity (*Q*_t_, mg g^−1^), was calculated as (eqn (2)):^25^2
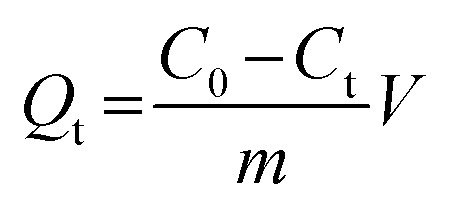


### 2.6 Application for analysis of DES in lake water samples

The Fe_3_O_4_@SiO_2_@APBA/MIPs were applied to extraction and then analysis of DES from lake water samples using the MSPE method coupled with HPLC.^24^ The process is illustrated in Fig. 1B. First, 2000 mL of water samples collected from Moon Lake located in Beijing University of Technology (China) were filtered with a 0.45 μm filter membrane three times under vacuum. Then the filtered samples were stored at 4 °C for further experiments.^38^ Before the first use, MIPs or NIPs were conditioned sequentially by immersion in ethanol (3 mL), elution solution (3 mL), and ultrapure water (3 mL) for 3 min, respectively. Subsequently, 80.0 mg MIPs were dispersed in 500 mL of the filtered samples or standard aqueous solutions, and then shaken for 160 min at 298 K, to achieve complete adsorption. MIPs or NIPs were collected using a magnet. After the MSPE step, saturated MIPs or NIPs were washed in sequence with 5.0 mL of ethanol and water, and followed by 5.0 mL of elution solution, and then separated magnetically. Finally, the collected eluents were determined by HPLC.

## 3. Results and discussion

### 3.1 Optimizing preparation conditions for MIPs

The molar ratio of the template-functional monomer is important in a successful imprinting process because of its effect on the number of recognition sites formed in MIPs and the quality of the MIPs. Adsorption tests of Fe_3_O_4_@SiO_2_@APBA/MIPs prepared with different molar ratios (1 : 3, 1 : 4, and 1 : 5) were carried out at 298 K in 60 mg L^−1^ DES aqueous solution. The results indicated that the highest adsorption capacity of 18.85 mg g^−1^ was achieved at a ratio of 1 : 4, while only 40% and 65% were achieved at ratios of 1 : 3 and 1 : 5, respectively. Therefore, a molar ratio of 1 : 4 was adopted in subsequent experiments. Moreover, the amount of MIP coating on the surface of Fe_3_O_4_@SiO_2_ can also influence the adsorption capacity. Different quantities of Fe_3_O_4_@SiO_2_ (25, 50, 75, and 100 mg) were used for the preparation of MIPs with fixed amounts of the other reactant. The results demonstrated that 50 mg of Fe_3_O_4_@SiO_2_ was optimal for further study.

### 3.2 Characterization of Fe_3_O_4_@SiO_2_@APBA/MIPs

**Fig. 2:**
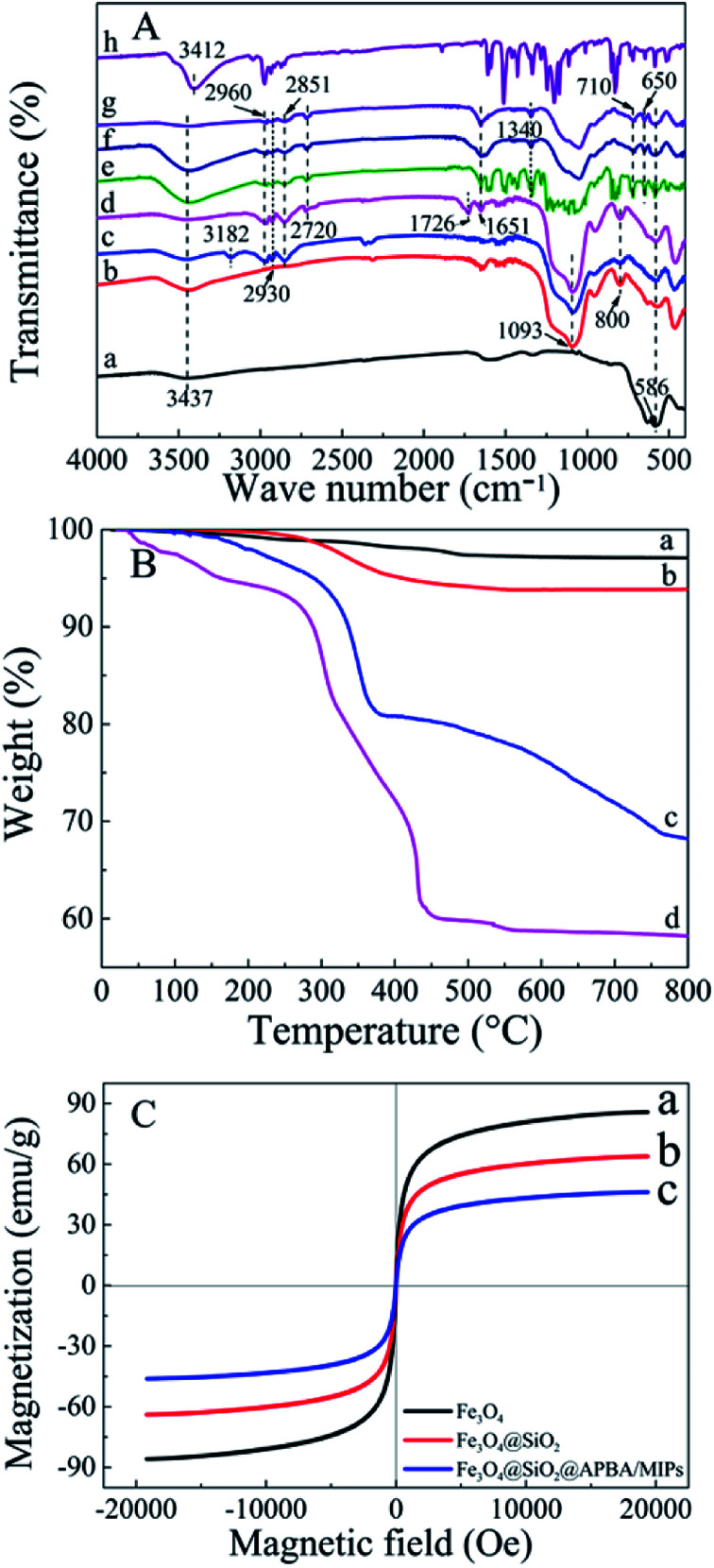
(A) FTIR spectra of (a) as-prepared Fe_3_O_4_, (b) Fe_3_O_4_@SiO_2_, (c) Fe_3_O_4_@SiO_2_–NH_2_, (d) glutaraldehyde-capped Fe_3_O_4_@SiO_2_, (e) Fe_3_O_4_@SiO_2_@APBA-DES/MIPs, (f) Fe_3_O_4_@SiO_2_@APBA/MIPs, (g) Fe_3_O_4_@SiO_2_@APBA/NIPs and (h) DES. (B) TGA curves of (a) Fe_3_O_4_, (b) Fe_3_O_4_@SiO_2_, (c) Fe_3_O_4_@SiO_2_@APBA/MIPs and (d) Fe_3_O_4_@SiO_2_@APBA/NIPs. (C) VSM magnetization curves of (a) Fe_3_O_4_, (b) Fe_3_O_4_@SiO_2_ and (c) Fe_3_O_4_@SiO_2_@APBA/MIPs.

The FTIR (KBr) spectra of the functionalized Fe_3_O_4_ and imprinted NPs are presented in Fig. 2A. For Fe_3_O_4_ (curve a), the strong absorption peaks located at 586 and 3437 cm^−1^ could be attributed to stretching vibrations of Fe–O and Fe–OH bonds. The bands around 1093 and 800 cm^−1^ were caused by asymmetric and symmetric stretching vibrations of the Si–O groups (curve b), demonstrating that covalent bonds between the silane coupling agent and magnetite surface were generated on Fe_3_O_4_@SiO_2_ NPs.^39^ Characteristic peaks at 2930 and 2851 cm^−1^ (stretching vibration of –CH_2_–), 2960 cm^−1^ (stretching vibration of –CH_3_) and 3182 cm^−1^ (corresponding to –NH_2_) could be ascribed to the APTES successfully modified on silica shells (curve c). The new bands at 1726 and 2720 cm^−1^ were, respectively, the characteristic of the C=O stretch and the C–H stretch of the aldehyde group from glutaraldehyde (curve d). Moreover, the disappearance of the –NH_2_ stretching vibration peak and the appearance of a new band of C=N at 1651 cm^−1^ demonstrated that glutaraldehyde was grafted onto the surface of Fe_3_O_4_@SiO_2_–NH_2_ by condensation with dehydration.^25,40^ Bands centered at 710 and 1340 cm^−1^ can be assigned to –B–OH bending and stretching vibrations of APBA, respectively (curves e–g). The new bands centered at 650 cm^−1^ might result from the C–B bond. Moreover, the increment of peak intensity at 1651 cm^−1^ from C=N, and the absent peak of C=O at 1726 cm^−1^ observed in Fig. 2A (e–g) can be attributed the contribution of APBA. The results proved that poly(APBA) was bound to the Fe_3_O_4_@SiO_2_ surface by covalent bonds. However, the expected bands around 586, 1093, and 1340 cm^−1^ corresponding to FeO, Si–O and Si–O bonds, respectively, in Fe_3_O_4_@SiO_2_@APBA-DES/MIPs were overlapped with new peaks at 500–1600 cm^−1^ (curve e). These new bands were in accordance with bands from the FTIR spectrum of DES (curve h). Furthermore, the shifted stretching frequency of the O–H group from 3437 to 3412 cm^−1^ (curve h) was due to the formation of hydrogen bonding between hydroxyl groups. After the sample was eluted, no characteristic bands of DES could be observed (curve f), indicating that the DES molecules were removed from the MIP composite. All results indicated that MIP layers were grafted on the surface of Fe_3_O_4_@SiO_2_ NPs.

Thermogravimetric analysis (TGA) curves for Fe_3_O_4_, Fe_3_O_4_@SiO_2_, Fe_3_O_4_@SiO_2_@APBA/MIPs and NIPs are shown in Fig. 2B. Weight loss at a temperature less than 200 °C can be attributed to the elimination of water. The weight loss for Fe_3_O_4_ NPs and Fe_3_O_4_@SiO_2_ NPs was approximately 1% (curve a) and 4% (curve b), respectively, when heated to 800 °C. The weight loss can be attributed to the decomposition of some contaminations and the grafted silane agent. Moreover, the weight loss of 25.9% for Fe_3_O_4_@SiO_2_@APBA/MIPs suggested that the imprinted polymers were grafted on Fe_3_O_4_@SiO_2_ (curve c). Significant weight loss for Fe_3_O_4_@SiO_2_@APBA/NIPs (35.8%, curve d) could be observed. The slight difference between the imprinted NPs and non-imprinted NPs may be due to the different grafting density caused by DES. The difference in thermal stability between these NPs showed that the imprinted polymers successfully grafted onto the Fe_3_O_4_.

The magnetic saturation test was performed at room temperature using a VSM to characterize the magnetic properties of Fe_3_O_4_ core-based nanoparticles. The three magnetic hysteresis loops with similar general shape are illustrated in Fig. 2C. As shown, the saturation magnetization of Fe_3_O_4_, Fe_3_O_4_@SiO_2_, and Fe_3_O_4_@SiO_2_@APBA/MIPs at a field of 2.0 × 10^4^ Oe decreased from 85.8 to 63.9, and then to 46.3 emu g^−1^, with increasing thickness of the modified layer on the surface of Fe_3_O_4_. However, the decrease did not clearly affect the magnetic performance of the Fe_3_O_4_@SiO_2_@APBA/MIPs particles; their rapid magnetic responsivity was demonstrated by the achievement of rapid adsorption within 5 s under an applied magnetic field. The adsorption experiments confirmed that MIP NPs can be used for effective magnetic separation.

**Fig. 3:**
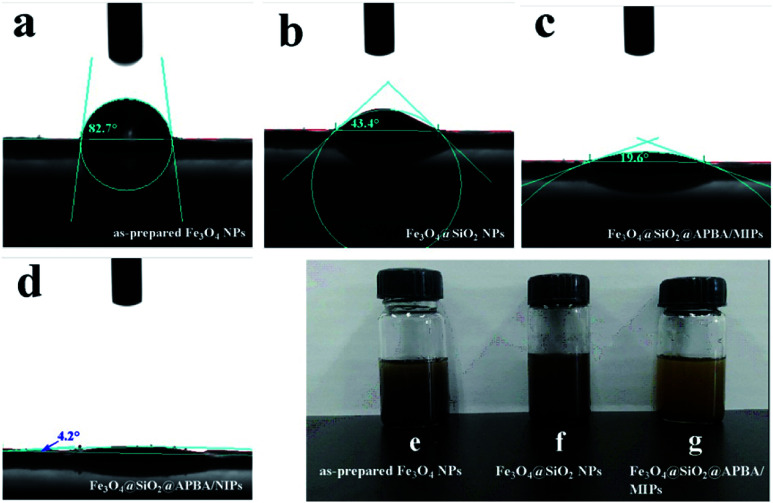
Static water contact angles of the as-prepared Fe_3_O_4_ NPs (a), Fe_3_O_4_@SiO_2_ NPs (b), Fe_3_O_4_@SiO_2_@APBA/MIPs (c), Fe_3_O_4_@SiO_2_@APBA/NIPs (d), and photographs showing the dispersion in pure water at 298 K of 1 mg mL^−1^ of the as-prepared Fe_3_O_4_ NPs (e), Fe_3_O_4_@SiO_2_ NPs (f), Fe_3_O_4_@SiO_2_@APBA/MIPs (g) after settling down for 24 h.

Water contact angle experiments were performed to accurately evaluate the surface hydrophilicity of the nanoparticles.^25,26^Fig. 3 shows the profiles of water droplets on compacted films of the Fe_3_O_4_ nanoparticles, Fe_3_O_4_@SiO_2_ nanoparticles, Fe_3_O_4_@SiO_2_@APBA/MIPs, and Fe_3_O_4_@SiO_2_@APBA/NIPs, respectively. The as-prepared Fe_3_O_4_ NPs were hydrophobic, with a contact angle of 82.7° (Fig. 3a). The static water contact angle of the Fe_3_O_4_@SiO_2_ NP film was 43.4° (Fig. 3b), indicating that Fe_3_O_4_@SiO_2_ NPs were more hydrophilic than Fe_3_O_4_ NPs because of the presence of rich polar functional groups on their surface. Furthermore, the contact angle of the Fe_3_O_4_@SiO_2_@APBA/MIP film was much smaller at 19.6° (Fig. 3c), demonstrating that the hydrophilic MIPs were successfully grafted onto the surface of the Fe_3_O_4_@SiO_2_ NPs. In addition, the contact angle of the Fe_3_O_4_@SiO_2_@APBA/NIPs was 4.2° (Fig. 3d), indicating their super hydrophilicity, which can be explained by the preparation through the grafting of APBA polymers directly onto Fe_3_O_4_@SiO_2_, with no DES. The improved surface hydrophilicity was beneficial to good dispersibility of the materials in water. As shown in Fig. 3(e) and (f), many more floats or sedimentations were present in Fe_3_O_4_ and silanized Fe_3_O_4_ suspensions than in the Fe_3_O_4_@SiO_2_@APBA/MIPs suspension. The excellent dispersion of Fe_3_O_4_@SiO_2_@APBA/MIPs in water provided greater opportunity for the DES molecules to access the imprinted cavities.

**Fig. 4:**
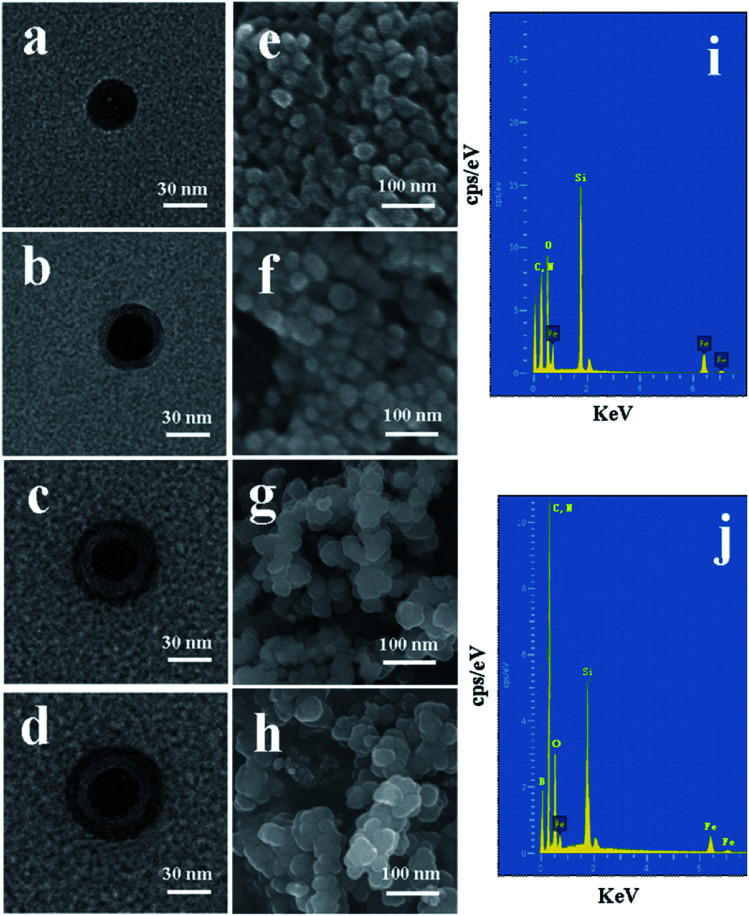
TEM images of (a) as-prepared Fe_3_O_4_, (b) Fe_3_O_4_@SiO_2_, (c) Fe_3_O_4_@SiO_2_@APBA/MIPs, and (d) Fe_3_O_4_@SiO_2_@APBA/NIPs. SEM images of (e) as-prepared Fe_3_O_4_, (f) Fe_3_O_4_@SiO_2_, (g) Fe_3_O_4_@SiO_2_@APBA/MIPs, and (h) Fe_3_O_4_@SiO_2_@APBA/NIPs. EDS spectra of (i) as-prepared Fe_3_O_4_@SiO_2_ and (j) Fe_3_O_4_@SiO_2_@APBA/MIPs.

The morphological structure and particle size of the synthesized nanoparticles can be observed by TEM and SEM. It can be observed from Fig. 4a–d that the mean diameter sizes of Fe_3_O_4_, Fe_3_O_4_@SiO_2_, Fe_3_O_4_@SiO_2_@APBA/MIPs, and Fe_3_O_4_@SiO_2_@APBA/NIPs were approximately 30, 40, 50 and 55 nm, respectively. An SiO_2_ shell with a thickness of approximately 5 nm was clearly seen to be uniformly coated over the Fe_3_O_4_ dark core (Fig. 4b), forming the first layer of the core–shell structure, indicating the success of the fully coated silica shell. After imprinting, another external polymer layer with a thickness of approximately 5 nm appeared around Fe_3_O_4_@SiO_2_ micro-particles (Fig. 4c), which suggests that the second imprinted shell had been successfully grafted.^41^ As seen from the SEM images, initially the Fe_3_O_4_ has a rough surface and a regular spherical shape, but relatively severe agglomeration (Fig. 4e). After grafting by the silane coupling agent, the agglomeration of Fe_3_O_4_@SiO_2_ was alleviated, the surface became slightly smooth, and the morphology of the sphere became more regular (Fig. 4f). The chemical composition and elemental mapping of Fe_3_O_4_@SiO_2_ and Fe_3_O_4_@SiO_2_@APBA/MIPs were characterized by X-ray EDS analysis. In the EDS spectrum of Fe_3_O_4_@SiO_2_ (Fig. 4i), the presence of iron, silicon, carbon, and nitrogen suggested that the silane coupling agent was grafted onto the surface of Fe_3_O_4_. After imprinting, the microspheres became larger, due to the coated imprinted polymers, and more uniform in size distribution (Fig. 4g). In addition, boron from APBA was observed in the EDS spectrum (Fig. 4j), while the peak for nitrogen overlaps with that of carbon. These results further confirm that imprinted polymers were coated on Fe_3_O_4_. For comparison, there was no remarkable difference in morphology and diameter between Fe_3_O_4_@SiO_2_@APBA/NIPs (Fig. 4d and h) and Fe_3_O_4_@SiO_2_@APBA/MIPs (Fig. 4c and g). Both MIP and NIP particles possessed similar uniform core–shell structures. However, the MIP NPs appeared to have a more uniform size distribution than the NIP NPs. There was a slight difference of 2.5 nm in the shell thickness of the NIPs compared with that of the MIPs (Fig. 4d), which might be due to the absence of DES molecules in the formation of the imprinting polymers shell. As shown, these results were in agreement with the above discussion on the FTIR spectra, static water contact angle, VSM, and TG analyses. Compared with other reports^34^ based on coprecipitation reactions or using the solvothermal method,^42^ the hydrophilic Fe_3_O_4_ magnetic nanoparticles synthesized in this paper possessed higher magnetization or smaller particle sizes, respectively. The encapsulation of Fe_3_O_4_ with a nonporous SiO_2_ shell could improve their dispersion in water, easily be modified with various groups, prevent the oxidization and agglomeration of Fe_3_O_4_, and then increase their reusability.^43,44^

### 3.3 Adsorption isotherm studies of Fe_3_O_4_@SiO_2_@APBA/MIPs

**Fig. 5:**
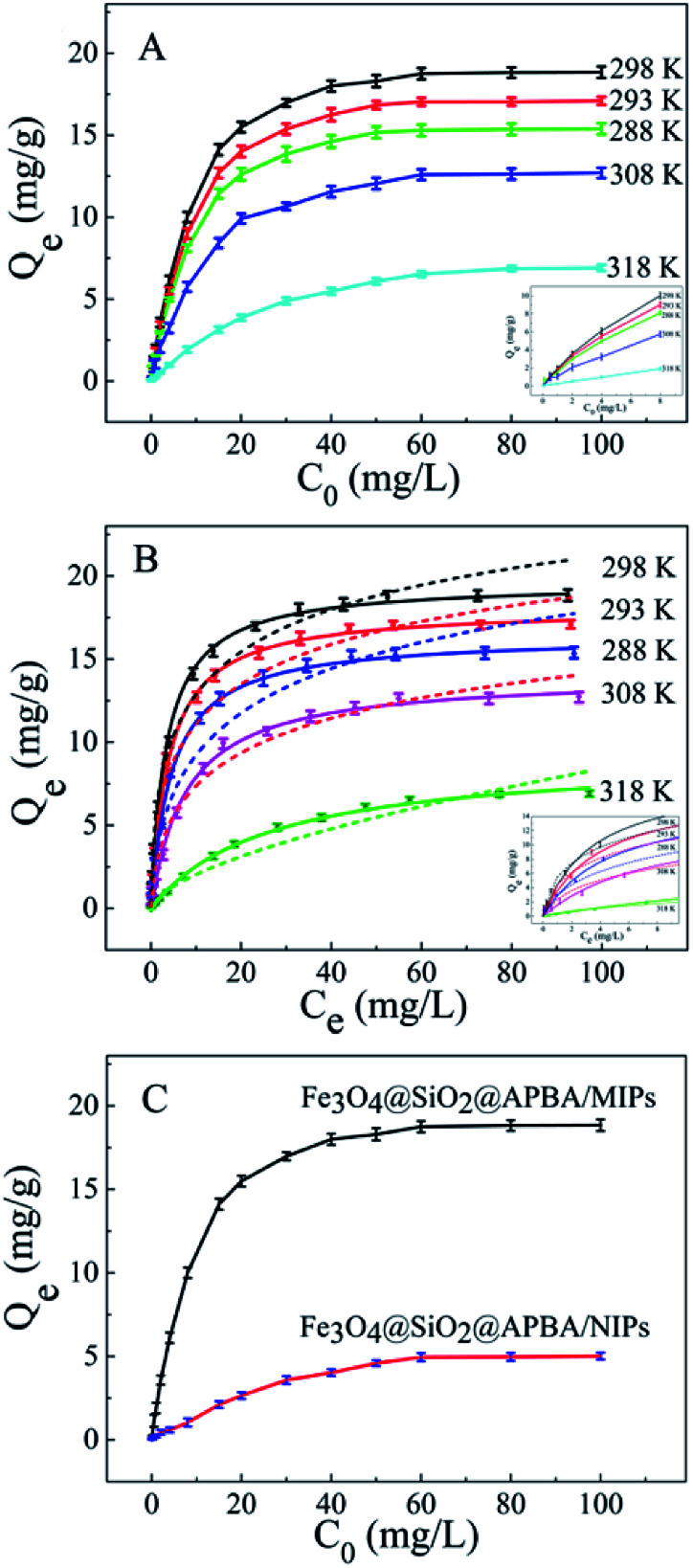
(A) Equilibrium adsorption isotherms of DES on Fe_3_O_4_@SiO_2_@APBA/MIPs at five different temperatures (20 mg of Fe_3_O_4_@SiO_2_@APBA/MIPs in 50 mL of DES solution shaken for 3 h); (B) fitted adsorption isotherms with two adsorption isotherm models; the solid line is the Langmuir model simulation and the dotted line is the Freundlich model simulation; (C) equilibrium adsorption curves of Fe_3_O_4_@SiO_2_@APBA/MIPs and Fe_3_O_4_@SiO_2_@APBA/NIPs for DES at 298 K. Inserts of (A) and (B): adsorption isotherms of DES solution with initial concentrations ranging from 0.0500 to 8.00 mg L^−1^.

The adsorption isotherms of DES on Fe_3_O_4_@SiO_2_@APBA/MIPs at five different temperatures are shown in Fig. 5A. The equilibrium adsorption capacities at 298 K were the highest among those at 288, 293, 308, and 318 K. When the temperature was higher than 298 K, the equilibrium adsorption capacities increased with decreasing temperature, which is consistent with previous findings showing that the imprinting cavities of MIPs prepared at low temperatures possess a similar three-dimensional structure at low temperature, such that MIPs are more effective at low temperatures.^45^ However, the capacities decreased with decrements in temperature below 298 K. The reason might be that the low temperature resulted in a slower diffusion rate of DES between the solution and the MIP film.^24^ Furthermore, it can be seen that the adsorption capacity of Fe_3_O_4_@SiO_2_@APBA/MIPs increased with increasing DES equilibrium concentration. The increase in DES concentration can accelerate the diffusion of DES molecules onto Fe_3_O_4_@SiO_2_@APBA/MIPs. Therefore, 298 K was chosen as the appropriate temperature for subsequent experiments.

The Langmuir and Freundlich adsorption isotherm models were used for the nonlinear fitting of experimental data and evaluation of the adsorption isotherms of Fe_3_O_4_@SiO_2_@APBA/MIPs (Fig. 5B). The Langmuir model is suitable for monolayer adsorption on uniform energy surfaces. The model equation is described in eqn (3):^46^3
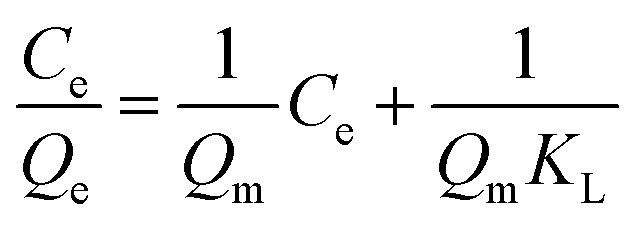
where *Q*_e_ (mg g^−1^) represents the equilibrium adsorption capacity of DES, *C*_e_ (mg L^−1^) is the equilibrium concentration of DES in solution, and *Q*_m_ (mg g^−1^) is the maximum adsorption capacity of the adsorbent. The Langmuir constant of *K*_L_ (L mg^−1^) is related to the affinity of the binding sites. The Freundlich model is suitable for multilayer adsorption occurred on heterogeneous surfaces. The model equation is expressed in eqn (4):^47^4
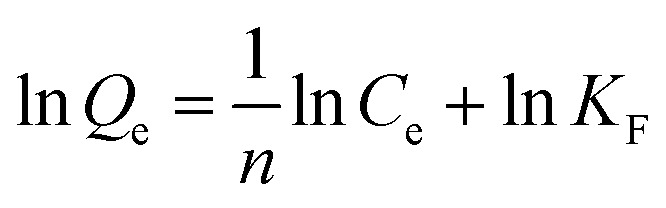
where the Freundlich constants *K*_F_ and *n* represent the adsorption capacity and adsorption favorability, respectively. Table 1 listed the fitted values of *Q*_m_, *K*_L_, *K*_F_, *n*, and *R*^2^ calculated from the above two nonlinear regression isotherm models. Adsorption isotherms of Fe_3_O_4_@SiO_2_@APBA/MIPs can be better described by the Langmuir model at five temperatures because their *R*^2^ values all exceed 0.99, and are much higher than those of the Freundlich model. Moreover, it is seen that the *Q*_m_ calculated from the Langmuir equation were very close to the *Q*_e(exp)_ values obtained from experiments.

**Table 1:** Adsorption isotherm parameters for DES adsorption on Fe_3_O_4_@SiO_2_@APBA/MIPs

Temperature	*Q*_e(exp)_ (mg g^−1^)	Langmuir			Freundlich		
		*Q*_m_ (mg g^−1^)	*K*_L_ (L mg^−1^)	*R* ^2^	*K*_F_	*n*	*R* ^2^
288 K	15.76	16.42	0.2167	0.9976	3.8734	2.2534	0.9594
293 K	17.48	18.08	0.2503	0.9990	4.4906	2.4193	0.9614
298 K	18.85	19.62	0.2917	0.9986	5.5139	2.7566	0.9721
308 K	12.65	14.04	0.1285	0.9989	2.4821	1.8384	0.9824
318 K	6.52	7.19	0.0382	0.9975	0.4953	1.6263	0.9691

Next, static adsorption experiments of NIP NPs were performed at 298 K for comparison with MIP NPs. At first, *Q*_e_ of the two adsorbents increased remarkably as the initial concentration increased from 0.500 to 50.0 mg L^−1^, and then reached saturation adsorption at 50.0 mg L^−1^ (Fig. 5C). However, *Q*_e_ of MIP NPs (18.85 mg g^−1^) was approximately 3.8 times of that of NIP NPs (4.96 mg g^−1^). These results suggest that NIP NPs have no specific adsorption properties.

### 3.4 Adsorption kinetic studies

**Fig. 6:**
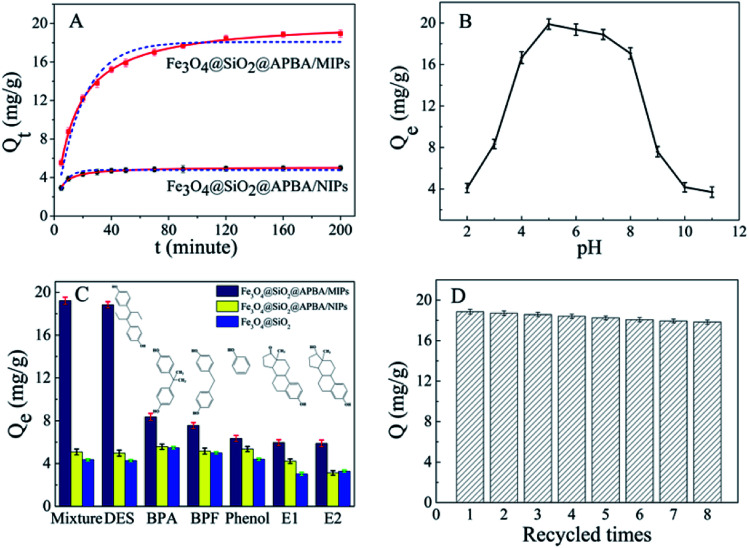
(A) Kinetic adsorption curves of DES on MIPs and NIP NPs at 298 K. The dotted line and solid line correspond to the pseudo-first-order and pseudo-second-order fitting, respectively. (B) Effect of solution pH on DES adsorption on MIP NPs. (C) Selective adsorption capacities of MIPs, NIPs, and Fe_3_O_4_@SiO_2_ NPs for DES, BPA, BPF, phenol, E1 and E2. (D) Regeneration cycles for MIP NPs.

The adsorption kinetics investigation showed that the minimum required time for the adsorption equilibrium for Fe_3_O_4_@SiO_2_@APBA/MIPs was 160 min at 298 K (Fig. 6A). From this finding, 160 min was chosen as the optimal extraction time. The adsorption process was quite fast in comparison with traditional imprinted materials which would take 12–24 h to reach equilibrium state.^48^ The reason for this can be attributed to the fact that the surface poly(APBA) imprinting films wrapped on Fe_3_O_4_@SiO_2_ nanoparticles provided more binding sites at their surface and achieved faster mass transfer. NIP NPs showed a similar trend, but with much lower adsorption capacities. Two kinds of adsorption kinetic models were applied to fitting the experimental kinetic data of MIP NPs for DES to study the rate control and mass transfer mechanism of the adsorption process of DES at Fe_3_O_4_@SiO_2_@APBA/MIPs according to previous reports.^38,39,42^ The pseudo-first-order model can be described as follows (eqn (5)):^49^

5 ln (*Q*_e_ − *Q*_t_) = ln *Q*_e_ − *k*_1_*t*

The pseudo-second-order model comprises all the steps of adsorption including external film diffusion, adsorption, and internal particle diffusion, and can be described as follows (eqn (6)):^50^6
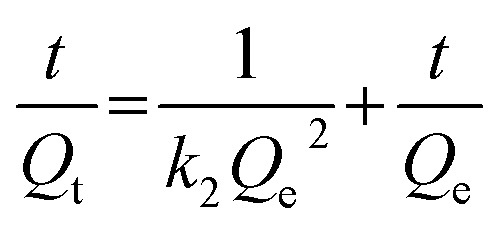
where *Q*_t_ (mg g^−1^) is the instantaneous adsorption amount at various times *t*, and *k*_1_ and *k*_2_ (min^−1^) are the adsorption rate constants. The plotted nonlinear regression fitting curves present a comparison of the two kinetic models (Fig. 6A). Corresponding fitting parameters and *R*^2^ are summarized in Table 2, and show that the pseudo-second-order model was a better fit for the higher regression coefficient *R*^2^ (>0.99, Table 2). Furthermore, the calculated adsorption capacity (*Q*_e(cal)_, Table 2) from the pseudo-second-order model agreed well with experimental data (*Q*_e(exp)_, Fig. 6A). Similar results have previously been reported for the adsorption of hydroxybenzoic acids on magnetic MIPs^42^ and estrogens on MIPs.^38^ Therefore, the pseudo-second-order model was more suitable for describing the mass transfer process of DES molecules on Fe_3_O_4_@SiO_2_@APBA/MIPs particles in solution. The adsorption process can be divided into three steps; *i.e.* boundary diffusion, intra-particle diffusion, and adsorption reaction. The complex effect of multiple adsorption mechanisms is suitable for adsorption process with saturation sites.^40^

**Table 2:** Parameters of the two adsorption kinetic models for DES on MIPs and NIP NPs

Absorbent	*Q*_e(exp)_ (mg g^−1^)	Pseudo-first-order			Pseudo-second-order		
		*R* ^2^	*k*_1_ (min^−1^)	*Q*_e(cal)_ (mg g^−1^)	*R* ^2^	k_2_ (min^−1^)	*Q*_e(cal)_ (mg g^−1^)
Fe_3_O_4_@SiO_2_@APBA/MIPs	18.85	0.9604	0.05446	18.08	0.9993	0.00364	18.83
Fe_3_O_4_@SiO_2_@APBA/NIPs	4.96	0.9399	0.17418	4.80	0.9939	0.05699	4.92

### 3.5 Effect of solution pH

The pH experiments were performed in 50 mL of 60 mg L^−1^ DES solutions with different pH values, with 20.0 mg of Fe_3_O_4_@SiO_2_@APBA/MIPs dispersed in them for adsorption for 160 min. The competitive adsorption experiments were carried out in 50 mL of suspension with 20.0 mg of Fe_3_O_4_@SiO_2_@APBA/MIPs or NIPs and 60 mg L^−1^ of DES, BPA, BPF, phenol, E1, and E2 for adsorption for 160 min.

Fig. 6B shows the adsorption capacities of Fe_3_O_4_@SiO_2_@APBA/MIPs toward DES at different pH values. The capacities in a broad pH range (pH 4 to 8) were attractive, although decreased rapidly at pH values lower than 4 or higher than 8. This is because the net charge of DES differs from that of the adsorbent at different pH values. When the pH value is greater than 8, DES molecules possesses a negative charge value because of the phenolic hydroxyl anions.^25^ Meanwhile, APBA, with its weak boric acid groups, may dissociate in the high pH range.^29^ Thus, electrostatic repulsion between negatively charged MIPs and DES might result in the decreased adsorptivity of MIPs. Furthermore, the amino protonation might occur to APBA when the pH value of the solution was less than pH 4. As a result, hydrogen bonds were partially broken between APBA and DES, resulting in the decreased adsorptivity of MIPs to DES.^25^

### 3.6 Binding selectivity for DES

Five reference compounds (BPA, BPF, phenol, E1, and E2) were used for the evaluation of binding selectivity. Fig. 6C demonstrates the clear differences in capacity for Fe_3_O_4_@SiO_2_@APBA/MIPs between DES and reference compounds. In contrast, Fe_3_O_4_@SiO_2_@APBA/NIPs and Fe_3_O_4_@SiO_2_ NPs exhibited similar and poor adsorption toward the six reference compounds.

To further demonstrate the selectivity differences between imprinted and non-imprinted polymers, the parameters including the imprinting factor (IF), defined as *Q*_MIPs_/*Q*_NIPs_, and the relative selectivity constant (SC), defined as IF_DES_/IF_analog_, were calculated.^20^ The larger IF value demonstrated that MIPs for the analytes exhibited a higher selectivity. As presented in Table 3, the values of *Q*_MIPs_ for DES and IF_DES_ were larger than those of the other five reference compounds, indicating that Fe_3_O_4_@SiO_2_@APBA/MIPs possessed relatively higher affinity for DES than those of its reference compounds. The similar *Q*_NIPs_ and SC values indicated that the adsorption of six compounds on Fe_3_O_4_@SiO_2_@APBA/NIPs was non-specific. The above results confirm that the imprinting process was successfully achieved, and that Fe_3_O_4_@SiO_2_@APBA/MIPs exhibits excellent recognition ability and high selectivity toward DES, even in the mixture of DES and five reference compounds with the same concentrations.

**Table 3:** Imprinting factors and relative selectivity constants of DES and analogs for Fe_3_O_4_@SiO_2_@APBA/MIPs and Fe_3_O_4_@SiO_2_@APBA/NIPs

Analyte	*Q*_MIPs_ (mg g^−1^)	*Q*_NIPs_ (mg g^−1^)	IF	SC
DES	18.85	4.96	3.80	—
BPA	8.31	5.59	1.49	2.55
BPF	7.59	5.18	1.47	2.59
Phenol	6.33	5.35	1.18	3.22
E1	5.96	4.22	1.41	2.70
E2	5.88	3.12	1.88	2.02

### 3.7 Reusability

The regeneration of the adsorbent is important in terms of practical applications. Saturated Fe_3_O_4_@SiO_2_@APBA/MIPs (20 mg) was regenerated following consecutive steps of rinsing with ethanol three times, eluting under repeated shaking, and finally washing thoroughly with ethanol and ultrapure water. Regenerated MIPs were reused to extract 60 mg L^−1^ of DES standard aqueous solutions, with the adsorption test was repeated over seven successive adsorption-regeneration recycles. The reusability was investigated by monitoring the adsorption capacity recovery. As shown in Fig. 6D, the adsorption capacity remained at 17.83 mg g^−1^ after seven recycling procedures, and the adsorption efficiency lost was only 5.4% compared with the initial capacity (relative standard deviation [RSD] = 2.0%, *n* = 8). This excellent reusability and stability may be attributed to the properties of high chemical stability and good magnetic separation of the core–shell magnetic MIP, as well as the rapid mass transfer process.

### 3.8 Extraction performance for DES

An HPLC method for DES was established by using the Fe_3_O_4_@SiO_2_@APBA/MIPs as adsorbents of MSPE. Different amounts of Fe_3_O_4_@SiO_2_@APBA/MIPs, ranging from 20 to 100 mg, were used to extract DES from 500 mL extraction solvent when 10 μg L^−1^ DES solution and 160 min of shaken-auxiliary extraction were adopted. The results show that recoveries could be higher than 95% when 80 mg adsorbent was used. However, when the amount of adsorbent was further increased, there was no clear increase in recovery. When the extraction time was increased from 30 min to 160 min, the recovery increased correspondingly from 45% to 95%. However, on further increase in extraction time, there was nearly no further increase in recovery. Therefore, shaken-auxiliary extraction for 3 h was adopted. Methanol, 0.1 M acetic acid, and different ratios of their mixture were tested as eluting solvents. The best recovery was obtained when 5 mL of a mixture of methanol-0.1 M acetic acid (9 : 1, v/v) was used.

**Fig. 7:**
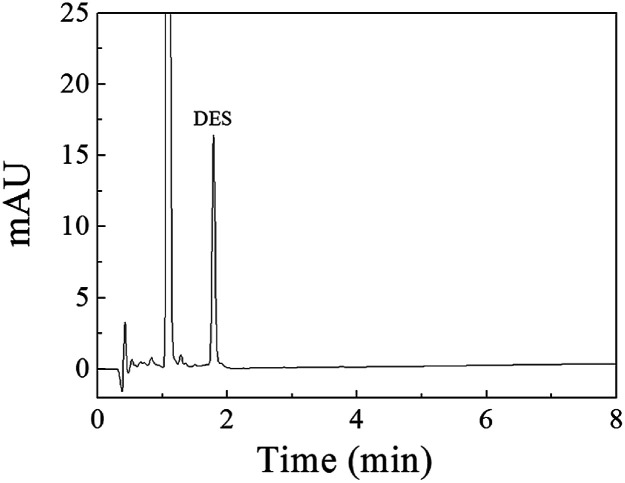
Chromatogram of the eluate obtained using 80 mg of Fe_3_O_4_@SiO_2_@APBA/MIPs to extract 500 mL of the DES standard solution (0.42 μg L^−1^) for 160 min at 298 K.

A linear regression analysis was performed to obtain the calibration curves for detection of DES, and the ratios of HPLC peak areas (*A*, mA U s) *versus* corresponding concentrations of DES (*C*, μg L^−1^) showed good linearity from 0.080 to 150 μg L^−1^ with correlation coefficients of *R*^2^ value of 0.9992. The regression equation was *A* = 49.6C − 2.8. The limit of detection (S/N = 3) was 0.03 μg L^−1^ DES. The chromatogram of the eluate obtained using Fe_3_O_4_@SiO_2_@APBA/MIPs to extract DES standard solution is shown in Fig. 7. The method accuracy was studied by examining recoveries of spiked water samples at three levels (1.0, 10, and 100 μg L^−1^), and the recovery values were in the range of 95.6–103.4%. The intra-day and inter-day precisions of the method were given by the calculated RSD of extraction and analyses of DES at different spiked concentrations. The spiked concentrations at the above three levels were performed on the same day six times per day and on different days for consecutive six days, respectively.^38^ The RSD values representing intra-day precision were 3.6%, 3.2% and 2.4% for the three concentrations, respectively (*n* = 6). The RSD for inter-day precision over 6 days were all less than 5.0% (4.8%, 4.2% and 3.8%, respectively, *n* = 6). Therefore, the results show that the proposed MSPE-HPLC method was applicable for rapid, sensitive, accurate, and quantitative determination of DES from water samples.

### 3.9 Method performance comparison

The proposed MIPs-MSPE-HPLC method for DES was compared with the other MIP-based pretreatment methods toward estrogens (Table 4). These reported methods were related to DES imprinted polymers synthesized and adsorption evaluated in organic solution, such as methanol,^6,13^ ethanol,^14,15^ acetonitrile,^1,17^ and chloroform.^16^ However, only a few studies on MIP adsorption for DES in aqueous phase have been reported.^5,27,51^ As seen from Table 4, compared with previous reports,^1,6,15,17,24^ the method in this paper not only created higher sensitivity, lower LODs and higher adsorption capacity to DES in aqueous solution, but also provided simple and fast pretreatment method.

**Table 4:** Analytical performances comparison of this MIPs-MSPE based on Fe_3_O_4_@SiO_2_@APBA/MIPs with other MIPs based methods for estrogens by HPLC^a^

Support	Functional monomer	Solvent	Evaluation solution	Target estrogens	Adsorption capacity (mg g^−1^)	Extraction method	Analytical method	Linearity range (μg L^−1^)	LODs (μg L^−1^)	Real sample	Ref.
RAFTPP	MAA, NIPAM	Acetonitrile	Aqueous	BPA	8.292	Packed SPE	HPLC	—	41.0 ng L^−1^	Seawater	24
—	MAA	Chloroform	Methanol	DES	8.43	MISPE	HPLC-DAD	—	1.8	Seawater	6
Silica gel	APTES	Methanol	Methanol	DES	62.58	SPE	HPLC	—	60	Fish samples	13
ATP	AA, MBAA	Ethanol	Ethanol	DES	105.14	SPE packing	HPLC	—	3	Pond water, fish samples	14
Silica-coated Fe_3_O_4_	APTES, PTMOS	Ethanol	Ethanol	DES, E3, E2	6.57, 5.38, 3.82	SPE	HPLC	0.3–100	0.08–0.27	Lake, river water	15
CNTs@SiO_2_	Silica	Acetate buffer	Chloroform	DES, E3, E1	30.46	Removing	HPLC	50–1 × 10^4^	10.2–16.1	River, lake, tap water	16
Fe_3_O_4_@nafion	Dopamine	Tris–HBS	Aqueous	DES	9.74	DSPE	HPLC-DAD	3.5–1000 ng g^−1^	0.99 ng g^−1^	Milk	5
Fe_3_O_4_@SiO_2_	MAA	Acetonitrile	Acetonitrile	DES	3.086	MMIPs	HPLC-UV	20–8000	3.6/6.3	Pond water, milk	17
HFT	MAA	Acetonitrile	Acetonitrile	DES	—	Microextraction	HPLC	7.5–200	2.5	Milk	1
M-ATP	CyD	Aqueous	Aqueous	E1, E2, E3, DES	0.04–0.1 mmol g^−1^ 10.73 (DES)	On-line SPE	HPLC-UV	30–2500 ng g^−1^	1–8 ng g^−1^	Milk	27
Fe_3_O_4_@SiO_2_	AA	MeOH, ACN	Aqueous	E1, E2, E3, DES	0.216–0.16	SPME	HPLC-UV	8–2000 ng g^−1^	1.5–5.5 ng g^−1^	Milk powder	51
Fe_3_O_4_@SiO_2_	APBA	Aqueous	Aqueous	DES	18.85	MSPE	HPLC-DAD	0.08–150	0.03	Lake water	This work

a PP: precipitation polymerization; MAA: α-methacrylic acid; NIPAM: *N*-isopropyl acrylamide; MISPE: molecularly imprinted solid-phase extraction; ATP: attapulgite nanofibrillar clay; MBAA: *N*,*N*-methylene-bisacrylamide; AA: acrylamide; PTMOS: phenyltrimethoxysilane; CNTs: carbon nanotube; Tris–HBS: Tris–HCl buffer solution; HFT: hollow fiber tube; M-ATP: methylacryloxypropyl modified attapulgite; CyD: acryloyl-β-cyclodextrin; SPME: solid-phase microextraction.

### 3.10 Applications

**Fig. 8:**
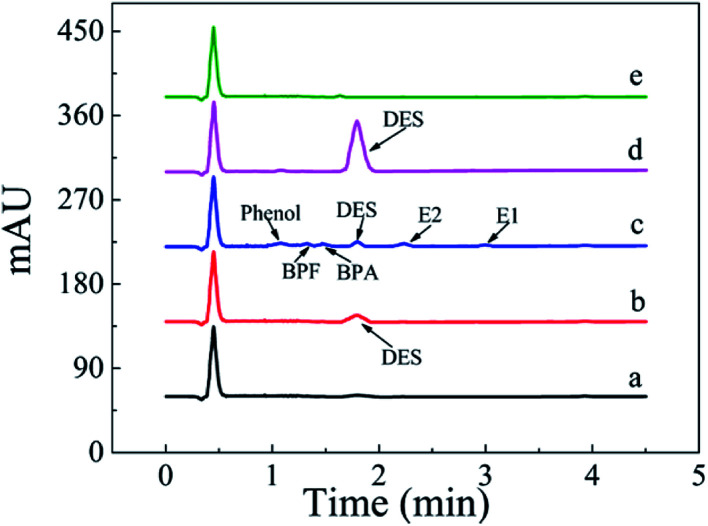
HPLC-DAD chromatograms of the lake water sample. From the bottom to the top: (a) lake water sample without any pretreatment; (b) non-spiked sample extracted with Fe_3_O_4_@SiO_2_@APBA/MIPs; (c) lake water sample spiked with DES, BPA, BPF, phenol, E1, and E2 individual at 1.5 μg L^−1^ without extraction; spiked samples extracted for 160 min at 298 K with 80 mg of (d) Fe_3_O_4_@SiO_2_@APBA/MIPs or (e) Fe_3_O_4_@SiO_2_@APBA/NIPs.

The MSPE-HPLC method was applied to determine DES in lake water samples. There was almost no DES peak in HPLC-DAD chromatogram obtained from natural sample without enrichment or spiking (Fig. 8a). However, DES in the sample could be detected when analyzed by MSPE-HPLC based on Fe_3_O_4_@SiO_2_@APBA/MIPs under optimized conditions, and the concentration value was 0.08 μg L^−1^ (Fig. 8b). Then the sample was spiked several times with 1.5 μg L^−1^ standard solutions of DES, BPA, BPF, phenol, E1, and E2, and the peak signals of analogs were all very weak (Fig. 8c). Therefore, quantitative analysis of trace DES in spiked samples by HPLC method without selective pretreatment process was difficult. After being enriched and extracted by MSPE based on MIPs and NIPs respectively, DES can be selectively adsorbed and then concentrated remarkably (Fig. 8d), and the peak of DES appeared distinctly. No obvious DES peak was observed in the eluted solution from NIPs (Fig. 8e), which also demonstrated the selectivity effect of the MIPs. Furthermore, the enrichment factor calculated was approximately 1900 for DES. The value of the enrichment factor demonstrated that Fe_3_O_4_@SiO_2_@APBA/MIPs possessed high pre-concentration ability for DES (Fig. 8d).^38^ The lake water sample was then spiked at three levels (0.100, 1.50 and 10.0 μg L^−1^) to validate the accuracy of the method in practical applications. Satisfactory recoveries of 97.1–103.2%, with RSD ranging from 2.8 to 4.3% (*n* = 6), were obtained (Table 5). The results indicated that the developed MIPs were ideal extraction adsorbents for MSPE, and thereby the proposed MIPs-MSPE-HPLC method was potentially applicable for highly efficient extraction and trace-determination of DES in real aqueous samples.

**Table 5:** Results of the determination of DES in the spiked lake water sample by HPLC method

Sample	Detected (μg L^−1^)	DES added (μg L^−1^)	Found (average ± SD) (μg L^−1^)	RSD (%, *n* = 6)	Recovery (%, *n* = 6)
Lake water	0.08	0.1000	0.1858 ± 0.0052	2.8	103.2
		1.5000	1.5740 ± 0.0614	3.9	99.6
		10.0000	9.7877 ± 0.4249	4.3	97.1

## 4. Conclusions

In this study, novel MIPs with excellent molecular recognition abilities and super water-compatibility (water contact angle of 19.6°) for the specific adsorption of DES in the aqueous phase were successfully prepared. The Fe_3_O_4_@SiO_2_@APBA/MIPs showed excellent features, such as high adsorption capacity (up to 18.85 mg g^−1^ at 298 K), rapid rebinding kinetics (only 160 min for adsorption equilibrium), good selectivity (imprinting factor of 3.80) and stability, as well as simple rapid magnetic separation. It was proven that Fe_3_O_4_@SiO_2_@APBA/MIPs provides great potential for pre-concentration of analyte samples in an environmentally friendly manner.

## Conflicts of interest

There are no conflicts to declare.

## Supplementary Material
